# Health Professionals on Cross‐Sectoral Collaboration Between Mental Health Hospitals and Municipalities: A Critical Discourse Analysis

**DOI:** 10.1111/nin.12685

**Published:** 2024-11-19

**Authors:** Kim Jørgensen, Kristine Bro Jørgensen, Jesper Frederiksen, Emma Watson, Morten Hansen, Bengt Karlsson

**Affiliations:** ^1^ Department of Nursing and Health Promotion, Faculty of Health Sciences Oslo Metropolitan University Oslo Norway; ^2^ Department of People and Technology Roskilde University Roskilde Denmark; ^3^ Mental Health Center Glostrup, Capital Region Psychiatry Glostrup Denmark; ^4^ Medical Department Zealand University Hospital Roskilde Denmark; ^5^ Department of Regional Health Research University of Southern Denmark Odense Denmark; ^6^ Nottinghamshire Healthcare NHS Foundation Trust Nottingham UK; ^7^ Bostedsteamet Ishøj Denmark; ^8^ Department of Health, Social, and Welfare Studies, Faculty of Health and Social Sciences University of Southeastern Norway Drammen Norway

**Keywords:** cross‐sectoral collaboration, discourse analysis, mental healthcare, patient‐centered care, power dynamics

## Abstract

This study investigates the role of language in cross‐sector collaboration between mental health hospitals and municipalities, focusing on the challenges of maintaining continuity of care and integrating patient‐centered approaches. Using Fairclough's framework for critical discourse analysis, we examined focus group interviews with 21 healthcare professionals, including nurses, social workers, and psychiatrists, to identify key themes and patterns in how cross‐sector collaboration is discussed. The analysis revealed a dominant medicalized discourse in hospital settings, which often emphasized structured care processes like treatment plans and medication management, overshadowing more flexible, patient‐centered approaches common in community‐based services. Power dynamics were evident, with hospital professionals frequently positioned as active agents, while patients and community‐based workers were portrayed in more passive roles. Although efforts to involve patients in decision‐making were noted, these were often controlled by professionals, reflecting a mediated approach to patient empowerment. The findings highlight the cultural and structural divides between hospital and community services and suggest the need for improved communication strategies, integrated care pathways, and a shift toward more inclusive, patient‐centered care models. Addressing these discursive barriers is crucial for achieving more effective, integrated, and patient‐centered care, ultimately improving outcomes for patients.

## 1 Introduction

This research investigates the role of language as utilized by healthcare professionals in managing and articulating the complexities of collaboration across mental health hospitals and municipal services. It critically examines the concept of discourse and its impact on shaping and defining professional boundaries, reinforcing compartmentalized (siloed) thinking, and mediating between uniform approaches and tailored care in mental health services. According to Norman Fairclough, discourse is not merely the use of language in speech or writing but a form of social practice that shapes and is shaped by social structures (Fairclough 1989, 2013). Fairclough's perspective highlights that discourse both constructs the social world and is constructed by it. It involves the interplay of texts, social relations, and systems of knowledge and belief. In this context, analyzing discourse in healthcare allows us to understand how language functions not just as a vehicle of communication but as a powerful tool in constructing realities of professional practice, influencing organizational structures, and defining the roles and interactions among different healthcare sectors.

In this paper, we distinguish between “language” as a structured system of symbols used by professionals and “communication” as the broader process by which these symbols are exchanged and interpreted. While language forms the building blocks of discourse, communication involves the interactional process through which meaning is constructed, negotiated, and shared in cross‐sectoral collaborations.

Over the past few decades, the integration and collaboration of health services in the Global North have faced significant challenges. Research highlights the complexities and contradictions of collaboration, often rooted in structural and cultural differences across healthcare sectors. The use of language plays a critical role in these dynamics, as the way health professionals (professionals) communicate about and within collaborations can either bridge or exacerbate these divides. Even in nations with robust policy frameworks, fragmented service delivery, and insufficient collaboration persist, often manifested through misalignments in discourse among professionals. This leads to disjointed care and unmet needs for patients, emphasizing the need for a deeper understanding of linguistic practices in shaping healthcare collaborations (Andersen et al. 2017; Jørgensen et al. 2023). The Danish healthcare system struggles with effective cross‐sectoral collaboration due to structural changes aimed at restructuring health services (Ministry of the Interior and Health 2023, 2024). These changes have introduced new roles for professionals and redistributed tasks among various actors. As governments, local policymakers, and healthcare professionals continue to grapple with establishing coherent patient pathways, the challenges are exacerbated by increased specialization, an aging population with multiple chronic conditions, and mounting pressure to improve healthcare efficiency. The complexity of establishing collaboration and coordination among various sectors within the Danish healthcare system is further complicated by the use of language, which plays a crucial role in how these new roles and tasks are understood and enacted. Effective communication and clear discourse are essential for navigating these transitions and enhancing intersectoral collaboration (Burau et al. 2019). In mental healthcare, patients often encounter communication and coordination gaps between different hospitals and municipalities, leading to lost information and disrupted care continuity (Jørgensen, Andreasson, et al. 2022; Jørgensen et al. 2023). Ideally, patients should experience coherent and continuous care when transitioning from mental health hospitals to follow‐up care in municipalities (Association of Municipalities and the Government and Regions 2021). However, collaboration is hindered by a lack of common understanding and implementation of collaborative concepts in clinical practice (Abela‐Dimech and Vuksic 2018; Accordini et al. 2017; Mathisen et al. 2016). Treatment, care, or rehabilitation plans created in one sector are not effectively utilized in the next, requiring patients to restart their progress when transitioning between providers. Professionals also struggle to agree on the interpretation of mental and social challenges faced by patients, resulting in no shared starting point for facilitating cross‐sectoral collaboration (Jørgensen, Rasmussen, et al. 2022; Kelly et al. 2018; Larsen, Jensen, and Pedersen 2020; Olasoji et al. 2019).

Furthermore, research points to differences in the habitual basis of professionals' action that maintains institutional distinctions between mental healthcare and social psychiatry. Mental healthcare focuses on the clinical management of mental disorders, emphasizing diagnosis and treatment primarily through medication and psychotherapy. It typically occurs in healthcare settings like hospitals and clinics. In contrast, social psychiatry considers the broader social context of mental health, advocating for community‐based care and emphasizing the role of environmental and cultural factors. It promotes social integration and preventive outreach to enhance public understanding and reduce stigma. While mental healthcare targets the biological aspects of disorders, social psychiatry addresses social determinants, advocating for an integrated approach that combines both effective management and prevention of mental health issues (Frederiksen, Dahl, and Jørgensen 2020).

Cross‐sectoral network meetings have been implemented in mental health services within the Capital Region of Denmark. Mandated by management, these meetings aim to enhance collaboration across sectors and prioritize the recovery process of patients (Jørgensen, Rasmussen, et al. 2022; Jørgensen, Rasmussen, et al. 2020). Social workers typically organize these meetings, inviting patients, families, and relevant professionals from both hospitals and the community. These meetings bring together professionals and patients to develop a recovery‐oriented plan following discharge from a mental health hospital (Mental Health Region Copenhagen 2023). The objective is to support patients in their recovery process and promote coherence and coordination based on their goals and preferences (Biringer et al. 2016; Davidson et al. 2008; Pals and Hempler 2018; Ramon 2018; Topor, Boe, and Larsen 2022; Vaeggemose et al. 2018). However, professionals often face difficulties implementing the vision of recovery‐oriented network meetings due to a managerial focus on pragmatic aspects like medication management and social issues. In these cases, patients may find themselves as passive spectators in a paternalistic decision‐making structure (Brekke, Ness, and Lien 2018, 2020). Additionally, professionals do not always share a common understanding of recovery, with some viewing patients’ problems as symptoms to be treated, while others prioritize individual perspectives and support needed for recovery (Biringer et al. 2017; Borg, Karlsson, and Stenhammer 2013; Ness et al. 2014). “Recovery” in mental health is a particularly contested construct, embodying a spectrum of meanings from clinical recovery to a broader, more inclusive understanding that encompasses social integration and personal fulfillment (Davidson et al. 2008). This variability in definition affects how services are structured and delivered, often leading to gaps between theoretical ideals and practical execution. Similarly, “collaboration” is not just a practice but a complex interaction shaped by the sociopolitical contexts and professional discourses of those involved. Recovery‐oriented collaboration across mental health hospitals and community mental healthcare is fundamental to mental health services and a goal within government health policies in Scandinavia and other Western countries (Hoej et al. 2020). Rather than viewing patients as isolated cases with diagnoses and symptoms, professionals should focus on their strengths and aspirations for a fulfilling life. However, implementing this vision is challenging due to management frameworks prioritizing practical matters and a lack of a common understanding of recovery (Jørgensen, Andreasson, et al. 2022; Jørgensen et al. 2023; Jørgensen, Rasmussen, et al. 2020).

This study rigorously examines the complex interplay between language and practices of cross‐sectoral collaboration within mental health services. By concentrating on discursive practices, it endeavors to elucidate how professional discourse simultaneously mirrors and constructs the realities of integrated care and the delineation of professional roles within a disjointed healthcare framework. The investigation posits that the strategic use of language by professionals not only reflects underlying norms and values but actively shapes the operational dynamics of health service delivery.

Grounded in the theoretical premises of Critical Discourse Analysis, this research interrogates how linguistic choices influence perceptions and interactions among health professionals, thereby affecting the efficacy of cross‐sectoral collaboration. It aims to unravel the nuanced ways in which discourse serves as a conduit for both reinforcing existing practices and fostering transformative approaches to mental healthcare. By exploring the discursive mechanisms at play, this study seeks to propose strategies for optimizing communication that could bridge the existing divides and enhance collaborative efforts.

This inquiry is crucial for enhancing our comprehension of the pivotal role that language and discourse occupy in configuring healthcare practices and influencing policy implementations. Through a meticulous dissection of communication nuances among healthcare professionals, the research aims to illuminate pathways for more effective discourse strategies that could lead to improved patient outcomes and more coherent and integrated care trajectories. The guiding research question for this scholarly endeavor is as follows: How does language influence the practices and perceptions of cross‐sectoral collaboration among professionals within mental health services in Denmark?

## 2 Methodology

The use of (Fairclough 2013) analytical framework for examining discourse on cross‐sectoral collaboration is based on its ability to unpack complex linguistic structures, thereby providing a clear and organized analysis of discourse. This approach demystifies intricate language, allowing for a more nuanced exploration of how cross‐sectoral collaboration is discussed. It offers a systematic method for analyzing language, enabling a deep dive into the subtleties of discourse. Fairclough's framework also facilitates profound interpretative insights, revealing how language shapes perceptions and attitudes toward cross‐sectoral collaboration, akin to solving a puzzle to gain a comprehensive understanding (Beedholm, Lomborg, and Frederiksen 2014).

A social constructivist perspective underpins this analysis, recognizing that cross‐sectoral collaboration is fundamentally tied to social interactions, where experiences are formed and understood within a social context. This perspective aligns with Fairclough's discourse theory, which posits that discourse is not only a medium through which social relations are expressed and negotiated but also a form of social practice that shapes and is shaped by these relations. In this view, cross‐sectoral collaboration is inherently subjective and unfolds within a dynamic social environment, where the meanings and understandings are continuously constructed and reconstructed through discourse. Our cognitive processes, according to this framework, are not merely passive reflections of reality but are actively constructed through interactions that are mediated by language, emphasizing the central role of communication in shaping our perceptions and actions (Fairclough 2008, 2013). Discourses surrounding cross‐sectoral collaboration are deeply embedded within a complex web of social practices shaped by specific political, socio‐cultural, economic, and historical contexts (Middleton and Uys 2009; Winther Jørgensen and Phillips 2002). This research employs critical discourse analysis to reveal the linguistic and discursive elements underlying these social and cultural phenomena, especially as they evolve in the late modern era. While cross‐sectoral collaboration might appear straightforward, it often masks deeper layers of social influence that are not immediately apparent (Fairclough 2008, 1989,; Wenneberg 2000; Winther Jørgensen and Phillips 2002). This study explores how discourses are utilized to sustain social structures and relationships, which are closely intertwined with power dynamics (Fairclough 1989). Discourses do more than just convey language and narratives about the world; they also reflect and influence broader social practices. According to Fairclough, discourses not only represent the social world but also actively shape it, continually evolving through their interaction with social practices (Fairclough 1992, 2013).

### 2.1 Sample

To gain a thorough understanding of healthcare professionals' participation in cross‐sector collaboration within mental health services, we employed purposive sampling (Ames et al. 2019). We obtained informed consent from a diverse group of professionals, including primarily nurses, assistant nurses, occupational therapists, peer workers, pedagogues, and social workers, ensuring representation from multiple sectors within the mental healthcare system. Pedagogues in this context refer to specialists in educational and social practices who often work within therapeutic settings to support personal and social development. Each professional group received detailed information about the study during individual meetings. The research was conducted at a large mental health hospital in Region Zealand, Denmark, covering three inpatient wards and two outpatient departments. A total of 21 participants consented to participate in the study. While this study primarily sourced data from professionals at a mental health hospital, the inclusion of participants from broader sectors would potentially enrich the understanding of cross‐sectoral collaborative practices. Future research could extend to include health professionals from allied sectors such as primary care, social services, and community organizations to provide a more comprehensive view of the discursive practices surrounding collaboration in mental healthcare (see Table 1).

**Table 1:** The context of the participants.

Focus groups	Context	Number of participations	Title
A	An acute mental health unit	5	Three nurses and two assistant nurses
B	An acute mental health unit	2	A nurse and an assistant nurse
C	An acute mental health unit	5	Three nurses, an assistant nurse, and an occupational therapist
D	An outpatient unit	4	Two nurses, a peer worker, and a pedagogue
E	An outpatient unit	5	Three nurses, a social worker, and an occupational therapist
The number of participants		21	

### 2.2 Data Collection'

Focus group interviews were conducted within each mental health ward, engaging diverse groups of participants (Table 1), and these group discussions were recorded and transcribed to form the primary data for analysis. This approach allowed for a deep exploration of the participants' experiences with cross‐sectoral collaboration, generating rich and high‐quality data (Morgan 2012, 2014). Group interviews were particularly valuable for facilitating dynamic, interactive discussions where participants could build on each other's insights and reflect on their experiences more collaboratively. To ensure a comprehensive understanding of the topic, discussions were designed with both broad, open‐ended questions (Table 2) and more targeted probes informed by the existing literature (Olasoji et al. 2018; Waters et al. 2015). The open‐ended questions provided room for participants to express their perspectives freely, while the follow‐up questions helped delve deeper into specific aspects of cross‐sectoral collaboration.

**Table 2:** Interview guide.

Theme	Research questions	Interview questions
Introduction and Participant Background	Please begin by sharing your profession, years of experience in psychiatry, and the sector in which you are currently employed.	Can you tell us where you are currently employed and briefly describe your role?
Challenges in Cross‐Sector Collaboration	How do you experience collaboration between your sector and others involved in patient care (e.g., hospitals, community services, social services)?	Can you share an example of a successful cross‐sector collaboration? What made it effective?
		Can you provide an example where collaboration between sectors was difficult? How did this impact the patient's recovery process?
Coherence in the Patient Recovery Process	What challenges have you encountered when collaborating with other sectors to support a patient's recovery process?	What strategies or practices help maintain continuity of care as patients transition between different sectors?
Barriers to Coherent Care Pathways	How do you work with other sectors to create a coherent and continuous recovery pathway for patients?	Can you provide an example where the lack of coherence in care impacted a patient's recovery?
Adaptation and Individualization of Treatment Plans:	What factors hinder the creation of a seamless care pathway for patients as they move between different sectors?	What role does collaboration play in adapting treatment plans to ensure they are patient‐centered?
Limitations in Customizing Care Pathways	How do you and your cross‐sectoral colleagues tailor treatment plans to meet individual patient needs?	Can you share a situation where cross‐sectoral limitations affected your ability to adapt a treatment plan according to patient needs? What factors might limit your ability to fully consider individual patient preferences in collaboration with other sectors?

### 2.3 Data Analysis

Fairclough articulates that language usage within social practices configures discourse, which is defined by the specific linguistic choices employed in distinct contexts, thereby projecting meanings from particular viewpoints (Winther Jørgensen and Phillips 2002). He devised an analytical framework to examine how discourses as social practices not only constitute but are also constituted, suggesting a dialectical interplay with various social dimensions. In this vein, social practices should shape discourses, knowledge, identities, and social and power relations, which are in turn influenced by other social practices (Fairclough 1989, 1992, 2013). His critical discourse analysis aims to uncover how discursive practices maintain the social order and perpetuate power imbalances, advocating for an approach that illuminates and potentially rectifies these power dynamics to foster a more egalitarian society (Fairclough 1995, 2013).

Further, Fairclough proposed a tripartite model to guide empirical research in communication and society, advocating that the analysis of any communicative event encompass three analytical dimensions: (1) linguistic features of the text, (2) discursive processes involved in the text's production and consumption, and (3) the broader social practice context (Fairclough 1989, 1992, 2013; Winther Jørgensen and Phillips 2002).

Text analysis under this framework scrutinizes language's formal elements of vocabulary, grammar, syntax, and sentence structure to dissect how discourses and genres are constructed. Tools for this analysis include keyword emphasis, grammatical examination for conversational control, metaphor usage, and speaker affinity analysis. This methodical examination, including detailed line‐by‐line analysis, enhances the understanding of phenomena such as patient participation in mental healthcare through discursive representations.

In discourse analysis, the focus extends to understanding text creation and interpretation, exploring intertextuality, sources, and discourse shifts. As informed by Fairclough, our method involved analyzing “interdiscursivity” and “manifest intertextuality,” studying how texts are distributed and consumed, and assessing their coherence (Fairclough 1992). This comprehensive analysis provided insights into the broader social activities and engagements involved in the collaboration between mental health hospitals and municipalities, aligning with Fairclough's emphasis on the role of discourse within societal structures (Fairclough 2008). In addition, we lifted the social analysis by the understanding of habitus as the underlying schemes that guide the actions and language of the professionals in practice (Bourdieu 1990).

The data analysis was conducted by a multidisciplinary team of researchers, including a native English speaker and the first author, who is fluent in both Danish and English. The data were collected and transcribed in Danish, and the first author translated the transcripts into English. Given the study's focus on language, particular attention was paid to ensuring that the nuances of Danish constructs were accurately reflected in the English translation. The translation process involved an iterative approach. After the first author completed the translation, the text was reviewed in collaboration with the native English‐speaking researcher. This collaboration ensured that the translations were not only linguistically accurate but also conceptually aligned with the original data and the critical discourse analysis framework, which was developed primarily in English. While some Danish terms do not have direct English equivalents, careful consideration was given to preserving the cultural and social meanings embedded in the original language. The inclusion of a bilingual team allowed us to critically assess the impact of translation on the data analysis process. By incorporating the perspectives of both Danish and English speakers, we were able to ensure that the discursive practices analyzed remained faithful to the participants' original expressions. The reflection on language throughout the analysis process is crucial, as the study's focus on how language shapes cross‐sectoral collaboration requires careful attention to linguistic nuances across both languages. Finally, coding and thematic analysis were conducted on the translated data, ensuring that the key themes emerging from the focus group discussions were captured and analyzed within the framework of critical discourse analysis. The steps taken to ensure methodological rigor included regular team meetings, where the researchers discussed the findings and reviewed the consistency of the coding process.

### 2.4 Ethical Considerations

The research adhered to ethical standards in scientific inquiry, receiving approval from the Research Ethics Committee (Institutional Review Board) at Roskilde University, Denmark, under reference number KJ‐03.23. The study complied with the principles outlined in the Helsinki Declaration (The Ministry of Interior and Health 2017; World Medical Association 2013). As the research did not aim to exert physical or psychological influence on participants, formal permission from a biomedical ethics committee was deemed unnecessary. Participants were fully briefed on the project and provided both written and verbal consent to participate. The principal investigator elucidated the study's objectives and ensured participants comprehended their legal and ethical rights. Approval to collect empirical data was obtained from a manager at the Regional Zealand mental health organization. All invited participants willingly consented, and there were no withdrawals from the study.

### 2.5 Findings

In this section, we present our findings derived from the three‐dimensional analysis framework, guided by our research question: How does language influence the practices and perceptions of cross‐sectoral collaboration among healthcare professionals within mental health services in Denmark? This framework, as previously noted, encompasses text analysis, discursive practice, and social practice, allowing us to explore how language both reflects and shapes collaborative processes within the healthcare system. Through this approach, we analyze how healthcare professionals' discursive choices impact the understanding and implementation of cross‐sectoral collaboration (Fairclough 2008).

### 2.6 Language as a Tool for Shaping Social Norms in Healthcare

The language used by professionals is crucial in influencing social norms and actively contributes to shaping the societal landscape. It is in this context that the complex process of replicating and sustaining social and cultural dynamics occurs. According to Woods (2006), language in healthcare settings is shaped through discursive construction, influenced by various social and institutional factors. In settings such as mental health centers, conversations often focus on addressing challenges, diagnosing disorders, managing symptoms, providing support, and implementing therapeutic approaches. This underscores the critical role of communication skills in healthcare, where effective language use is essential for delivering care accurately and empathetically.

### 2.7 The Vocabulary of the Texts

When evaluating cross‐sectoral collaboration as discussed by health professionals, it becomes clear that the language they use actively constructs the concept of collaboration itself. Terms such as “patients,” “symptoms,” “treatment plans,” and “discharge” are not neutral descriptors but are part of the discursive process through which cross‐sectoral collaboration is produced and understood. Health professionals, in their discussions of collaboration, frequently refer to key terms like “network meetings,” “treatment plans,” “medication,” “residence,” and “discharge” to describe the mechanisms through which care is coordinated across sectors. These terms reflect the practical aspects of their work but also shape how collaboration is conceptualized and implemented. For example, “network meetings” are not just events but are constructed in discourse as essential spaces where collaborative efforts are organized. Similarly, “treatment plans” and “medication management” are discussed as central elements in ensuring continuity of care, yet the way they are framed by health professionals highlights the bureaucratic and medicalized nature of cross‐sectoral collaboration. By recognizing that terms like “network meetings” and “discharge” are socially constructed through the language of health professionals, we can better understand how collaboration is enacted in practice. These terms serve to create and sustain the processes that define collaboration between mental health hospitals and municipalities, illustrating that cross‐sectoral collaboration is something produced through the way professionals speak and interact.

### 2.8 Interactional Control in Healthcare: Grammar and Power Dynamics

In healthcare settings, interactional control refers to how language is used to manage the flow of communication and decision‐making, often reflecting underlying power dynamics. This concept is a crucial element of grammar in discourse, as it pertains to who controls the interaction, how turns in conversations are allocated, and who has the authority to speak or make decisions. Interactional control is particularly significant in the context of cross‐sectoral collaboration, where power imbalances between professionals and patients can influence outcomes. In network meetings, for example, professionals often dominate discussions by setting diagnostic criteria, outlining care plans, and determining treatment approaches. For example, “During our network meetings, it often feels like we have to navigate between professional decisions and patient involvement, but ultimately, the professionals hold the majority of control over the conversation.” These actions reflect a high degree of interactional control, where professionals exercise authority through their specialized knowledge and institutional roles. However, this control can marginalize the voices of individuals, families, and community representatives, who may have less influence over the decision‐making process. To illustrate this, sample excerpts from network meeting transcripts show how professionals lead conversations and make key decisions, with limited input from patients or other stakeholders.

Fairclough's framework of critical discourse analysis highlights how interactional control can either reinforce hierarchical structures or support a discursive trend toward democratization in healthcare. When professionals actively involve patients and community members in discussions, such as medication management or care plan evaluations, they reduce the asymmetry of power. This aligns with Fairclough's concept of “discursive democracy,” where control is distributed more equitably, and decision‐making processes become more inclusive.

For example, in discussions about medication management, professionals typically hold significant control over prescribing and monitoring. “We try to involve patients in decisions about their medication, but often, the final decision rests with us as professionals. It is a challenging balance to maintain.” However, by engaging patients in these conversations and considering their preferences, professionals can reduce their dominance, moving toward a more dialogic interaction. This interactional shift can help challenge traditional hierarchies, fostering a more balanced and participatory approach to healthcare decision‐making. The final evaluation of care plans presents another opportunity to reassess and balance power dynamics. By incorporating feedback from both professionals and patients, the process can ensure that the evaluation is not solely controlled by institutional authority but is co‐produced with those receiving care. This, in turn, reflects a move toward democratizing healthcare practices, as decision‐making becomes more collaborative and responsive to individual needs, thereby embodying Fairclough's principles of interactional control in discourse.

### 2.9 Transitivity

Transitivity, as it pertains to Fairclough's (1992) framework, involves understanding who the sender and receiver of messages in discourse are and how power dynamics manifest in these roles. In the provided interview, transitivity reflects the interactional control between professionals and patients, as well as among the professionals themselves. When discussing patient care, professionals often speak from an active position, detailing their actions and decisions (e.g., “I am contact person,” “we developed a treatment plan”). In contrast, patients are often described in more passive terms, as recipients of care (“the patient was admitted,” “the patient was discharged”). This transitivity highlights the power imbalance, with professionals positioned as the primary agents who dictate the course of events, while patients are positioned as subjects who experience these decisions. During the team discussions, there is a subtle shift in transitivity where professionals discuss collaborative decision‐making (“we work in teams,” “we discuss in the team”). This shift from individual to collective agency reflects a more distributed form of control, where decision‐making is shared among multiple professionals, reducing the hierarchical power structure to some extent.

A nurse from the inpatient ward comments on the importance of patient involvement, suggesting an attempt to shift some agency back to the patients (we need to ask them what a good course of treatment is). Here, the professionals acknowledge the value of including the patient's perspective, indicating a potential shift in transitivity where patients are positioned as active participants in their care. However, this is still mediated by the professionals, who decide when and how to involve the patients.

The interview reveals a complex interplay of transitivity, where professionals generally hold the active role of senders of messages, shaping the care process and patient outcomes. However, efforts are being made to include patients more actively in the discourse, reflecting a move toward more balanced power dynamics. Yet, the degree to which patients are truly empowered remains controlled by the professionals, underscoring the ongoing challenge of achieving equitable interactional control in healthcare settings.

### 2.10 Modality

Fairclough's concept of “modality” refers to how speakers express their attitudes toward the truth, necessity, or desirability of a proposition, reflecting their level of commitment, certainty, or authority. Modality is typically conveyed through modal verbs such as “can,” “must,” and “might,” or through adverbs that indicate degrees of obligation, permission, or probability. In the context of the provided transcript, modality plays a significant role in how participants negotiate their perspectives on patient care and professional collaboration. For example, when participants discuss meeting patients “where they are” and setting realistic goals, the use of modality reflects a cautious approach toward patient autonomy and the ethical responsibility of not imposing unrealistic expectations. This is evident when a nurse from an outpatient unit states, “it is no good setting a requirement to be a world champion in the 100‐meter dash if you can only run 10 meters.” The modality here softens the statement, showing an understanding and empathetic stance toward patient capabilities.

Modality also appears in discussions of patient involvement and decision‐making, where modal verbs convey possibilities and contingencies. For instance, another nurse from the outpatient unit states, “a good patient course is really when the patients and their relatives experience a high level of professionalism.” This use of modality indicates a desired, yet not absolute, outcome, reflecting the inherent uncertainties in patient care.

This use of modality also reveals the complex interplay between professional authority and patient empowerment. Professionals oscillate between asserting their expertise and acknowledging the limits of their authority when discussing patient autonomy and the importance of involving patients in decision‐making processes. This balance reflects a nuanced understanding of their roles within a recovery‐oriented framework, where they must guide patients without overstepping into paternalism. In this context, modality highlights the negotiation of power, knowledge, and responsibility between professionals and patients. It underscores the ethical tension between guiding patients and respecting their autonomy, illustrating how language is used to navigate these professional and moral complexities.

## 3 Discursive Practice

Discursive practice entails examining language to reveal interconnected discourse patterns that emerge in focus group interviews, emphasizing aspects of intertextuality and interdiscursivity. These discourses encompass “Professional Skepticism Discourse,” “Rule and Regulation Discourse,” “Interdisciplinary Collaboration Discourse,” “Patient Empowerment Discourse,” and “Cross‐Sectoral Coordination Discourse.”

### 3.1 Professional Skepticism Discourse

From the transcriptions of the focus group interviews, there is evidence of a Professional Skepticism Discourse. This can be seen in the way healthcare professionals express doubt or negative attitudes toward certain patients, particularly those who are frequently readmitted or viewed as “hopeless” cases. For instance, one participant stated, “We've tried everything with this patient, but nothing seems to work,” reflecting a sense of resignation and closure to further therapeutic interventions. This type of language signals a shift in focus from recovery to mere maintenance, reinforcing the belief that certain patients may not benefit from additional efforts.

This discourse also manifests in the way professionals discuss collaborative efforts. For example, during one focus group, a professional commented, “Engaging social services or vocational rehabilitation for this patient seems pointless; they've been through the system too many times.” This illustrates how skepticism can impede cross‐sectoral collaboration, as professionals may be less inclined to engage with other sectors or services, believing these efforts will be ineffective. The skepticism is not only reflected in individual care decisions but also impacts the broader collaborative framework within mental healthcare. For instance, professionals shared concerns about the potential success of integrated care, with one stating, “There's little point in coordinating with community services for some patients; they're not going to improve.” This demonstrates how skepticism can create barriers to effective collaboration, reinforcing siloed approaches where professionals hesitate to fully engage with other sectors.

Moreover, the Professional Skepticism Discourse appears to affect patients' perceptions of their care. Several professionals acknowledged that patients are often sensitive to the attitudes and beliefs of their caregivers. One participant noted, “You can see it in their faces they know when we don't believe in their recovery.” This statement suggests that professional skepticism can undermine patient motivation and engagement, further complicating the recovery process. Overall, the Professional Skepticism Discourse creates a cycle in which low expectations lead to reduced efforts from the care team, resulting in poorer outcomes for the patient. This, in turn, reinforces the original skepticism. The data clearly illustrate how this discourse hinders the possibility of a coordinated, holistic approach to care, which is essential in cross‐sectoral collaboration.

### 3.2 Rule and Regulation Discourse

The Rule and Regulation Discourse in mental healthcare, as articulated through the language of healthcare professionals, constructs the tension between institutional protocols and the provision of patient‐centered care. This discourse is produced through repeated expressions of frustration from professionals regarding the rigidity of institutional rules, which, while designed to ensure safety and consistency, are perceived as obstructing patient recovery and well‐being. For example, phrases such as “we have to follow the rules on patient movement” or “the visiting hours are strictly enforced” illustrate how professionals enact and reproduce the discourse of regulation, framing these rules as external constraints on their professional judgment and patient care.

Fairclough's notion of discourse as a social practice is evident here, as these professional expressions do not merely reflect individual frustrations but actively participate in maintaining and legitimizing the institutional power structures that prioritize order and safety over flexibility. The discourse positions professionals in a complex relationship with these rules: while they are obligated to follow them, the language used in interviews shows their discomfort and the perceived conflict between rule adherence and providing individualized care. For example, when a nurse explains that “we are bound by the policy, but it doesn't always work for the patient,” this reflects an implicit critique of the rule‐bound system that shapes the interaction between professionals and patients.

Within the Rule and Regulation Discourse, professional roles are constructed in a way that reveals friction in cross‐sectoral collaboration. Healthcare professionals describe scenarios where different sectors, such as inpatient wards, outpatient services, and social services are bound by conflicting regulations. The discourse surrounding these conflicts is evident in statements like “we follow one set of rules, and social services follow another,” which not only highlights the discursive construction of interprofessional and inter‐sectoral tensions but also reinforces the idea that rules can act as barriers to effective collaboration. This discourse reveals how the language used in the focus group interviews shapes perceptions and attitudes toward cross‐sectoral collaboration. Another key aspect of this discourse is its relationship to patient autonomy. The language used in professional discussions often positions patients as passive subjects of institutional control, particularly through the rigid enforcement of rules. Phrases like “patients must follow the hospital's policies” or “we restrict their movement for safety” illustrate how the discourse constructs patients as objects of institutional governance, with limited agency over their own care. The language used here reinforces the asymmetry of power between healthcare professionals and patients, where the former are positioned as the enforcers of rules, and the latter are cast in a passive role, restricted by institutional mandates.

However, moments of discursive resistance also emerge within this framework. For instance, when professionals suggest, “we need to involve the patients more in their care decisions,” they challenge the prevailing discourse by advocating for greater patient involvement and autonomy. This reflects Fairclough's concept of discursive struggle, where the dominant discourse is contested by alternative voices seeking to reframe the narrative around patient empowerment and individualized care.

### 3.3 Interdisciplinary Collaboration Discourse

In the discourse surrounding interdisciplinary collaboration in mental healthcare, significant emphasis is placed on integrating diverse professional backgrounds, such as nurses, social workers, psychologists, and psychiatrists, to deliver comprehensive care. This discourse aligns with Fairclough's concept of discourse practice, which posits that language shapes how professionals perceive and enact their roles. The portrayal of multiple disciplines collaborating underscores Fairclough's notion of interdiscursivity, where different discourses intersect to create new meanings and practices. The discussions reflect a recurring struggle between professional authority and patient involvement, highlighting the complexities of achieving balanced decision‐making in mental healthcare. For instance, a nurse might describe how their input on patient care routines is integrated with a psychologist's behavioral strategies to formulate more effective treatment plans. However, this discourse also illuminates several challenges, especially in aligning patient goals with treatment strategies across these disciplines. One nurse noted, “While we focus on patient comfort and daily care routines, sometimes there's a clash when treatment plans come more heavily from a psychiatric perspective focused mainly on medication.”

Such divergences in priorities highlight a critical issue Fairclough might interpret through the lens of power relations embedded within discourse. Psychiatrists often prioritize medication management, while social workers focus on addressing social determinants like housing, and psychologists emphasize therapeutic interventions. These differing views can lead to tensions, necessitating ongoing negotiation and compromise to maintain a unified approach to patient care. This struggle over whose perspective takes precedence reveals the power dynamics at play within interdisciplinary collaboration.

Another challenge highlighted in focus group discussions is the hierarchical nature of many healthcare settings, where the opinions of certain professions, such as doctors, are often more heavily weighted. This hierarchy can be understood through Fairclough's concept of ideology in discourse, where certain professional voices are given more authority, potentially hindering true collaboration and leading to disengagement. A social worker in a focus group mentioned, “Sometimes, it feels like the decisions have already been made before we even have our meetings.”

Logistical hurdles, such as coordinating schedules and communication across departments, also complicate collaboration. These challenges relate to Fairclough's textual analysis, where practical difficulties in collaboration are reflected in the discourse concerning the need for integrated care platforms and shared electronic health records. A nurse highlighted the difficulties of adopting new integrated care platforms: “We were asked to switch to the new digital record system, but many of us received limited training, making collaboration challenging.” While these tools are proposed to facilitate better collaboration, their success depends on proper implementation and training, reflecting a tension between the ideal and the practical realities of healthcare work. Moreover, the discourse underscores the importance of a shared understanding of the patient's needs and goals, integrating both clinical and cultural contexts. This patient‐centered approach ensures that the care provided is comprehensive and responsive to individual preferences, aligning with Fairclough's view of how discourse can shape social practices.

Finally, the discourse extends the concept of interdisciplinary collaboration beyond the immediate healthcare team to include partnerships with external sectors like social services and employment agencies. These external collaborations are crucial for addressing the broader social determinants of health that impact mental health outcomes. As one policymaker noted, “Integrating mental health services with social care is crucial but requires substantial coordination and mutual understanding.” This reflects Fairclough's notion of social practice, where discourse not only describes but also actively influences broader social and institutional practices.

### 3.4 Patient Empowerment Discourse

From the transcriptions of the focus group interviews, there is clear evidence of a Patient Empowerment Discourse, which emphasizes the involvement of patients in their care decisions. For example, one professional stated, “We need to ask the patient what a good course of treatment looks like for them,” highlighting the attempt to give agency to patients in shaping their treatment plans. This shows an active effort to involve patients as decision‐makers, reflecting the ideals of patient‐centered care. However, the discourse also reveals underlying tensions around professional control. In one instance, a healthcare provider mentioned, “The patient wanted to stop taking their medication, but we had to guide them back to the plan,” which demonstrates the challenge of balancing patient autonomy with professional responsibility. This evidence illustrates that while professionals strive to empower patients, there is often a fine line between empowerment and what Fairclough (2013) describes as “managed autonomy.” Patients may feel involved, but their choices are often shaped or limited by what professionals consider to be acceptable or safe. This is evident in multidisciplinary teams where conflicting sectoral perspectives, such as social services advocating for patients' rights vs. clinical settings prioritizing adherence to treatment create further challenges. For example, one social worker commented, “We sometimes clash with the healthcare team about what's best for the patient's recovery,” illustrating the power dynamics at play.

### 3.5 Cross‐Sectoral Coordination Discourse

From the transcriptions of focus group interviews, evidence of a Cross‐Sectoral Coordination Discourse emerges when professionals describe their efforts to manage patient transitions between mental health hospitals and community‐based services. For example, one participant mentioned, “Patients often get lost in the transition because the hospital follows a protocol, but community services are more flexible,” reflecting the power imbalance described by Fairclough (1989, 2013). This statement illustrates how the structured, institutional power of hospitals can overshadow the individualized, long‐term care provided by community services, creating mismatched expectations and inconsistent care delivery.

The interviews reveal that healthcare professionals often struggle with the lack of shared records and communication across sectors. One nurse noted, “We don't always get the full picture from the community side, and it delays our response,” indicating the fragmented nature of information‐sharing between sectors. This aligns with Fairclough, (1992) concept of transitivity, where the hospitals (active agents) dominate decision‐making while the community services (passive agents) react to upstream decisions.

This fragmentation is further highlighted when professionals discuss how patients “fall through the cracks.” As one participant expressed, “We had a patient discharged from the hospital, but the community services weren't prepared, and the patient ended up back in the emergency room.” This example shows the impact of siloed systems, where a lack of integration between medical, social, and psychological discourses, which Fairclough calls interdiscursivity leads to disjointed care.

The data clearly show that addressing these challenges requires a discursive shift toward an integrated care model. Fairclough's (2001) concept of recontextualization can be applied here: discourses from different sectors must be merged to create a cohesive care continuum. One example from the data illustrates this point: “We need to have regular interdisciplinary meetings where we all come to the table with the same information,” a professional remarked, indicating the necessity of collaborative protocols and shared care plans to align hospital and community efforts.

Through these discursive practices, professionals recognize the need for systemic changes. As one participant stated, “Our ultimate goal should be patient outcomes, not individual sector goals,” advocating for a patient‐centered approach that prioritizes continuity and integration across different sectors. This discourse illustrates the shift in mindset needed to reduce power imbalances and improve cross‐sectoral coordination, thereby reducing patient relapses and unnecessary hospitalizations.

## 4 Social Practice

In the context of Fairclough's framework, social practice refers to the broader social, cultural, and institutional contexts in which discourses are embedded and which they help shape (Fairclough 1992). Bourdieu's concept of habitus emphasizes how ingrained practices and dispositions influence behavior within these social contexts (Bourdieu 1977). In this study on cross‐sectoral collaboration between mental health hospitals and municipalities, social practice involves understanding how professionals' language reflects and reinforces existing power structures and institutional norms.

Fairclough's analysis highlights that language is actively involved in maintaining and transforming social practices (Fairclough 1995). For instance, the dominance of medicalized discourse in hospital settings perpetuates a hierarchical structure where certain forms of knowledge (medical expertise) are privileged over others (community‐based approaches) (Woods 2006). This positions hospital professionals as the primary authority, influencing collaboration dynamics and reinforcing power imbalances.

Bourdieu's concept of habitus helps us understand how these social practices are internalized by healthcare professionals. Habitus refers to ingrained habits, skills, and dispositions acquired through socialization within specific fields (Bourdieu 2010). In this study, the habitus of hospital professionals includes a preference for structured, protocol‐driven care, aligning with institutional emphasis on efficiency and standardization (Bourdieu 1990). This contrasts with community professionals in outpatient care, who often emphasize flexibility, patient‐centered care, and long‐term support. Evidence from focus groups indicates that community professionals often tailor care to individual needs and adapt interventions based on patient feedback, highlighting a more flexible approach compared to hospital protocols. These differing habitus shape practices and expectations, leading to a discursive divide between hospital and community care.

The interaction between these differing habitus creates tensions in cross‐sectoral collaboration, as professionals navigate conflicting demands. For example, the expectation that hospital staff adhere to strict protocols may clash with the adaptable approaches favored by community‐based workers, leading to difficulties in coordinating care and ensuring continuity for patients (Bourdieu 1986). These tensions are evident in the discourse used by professionals, where hospital staff may prioritize medical authority, while community professionals emphasize patient empowerment and individualized care.

Social practice also encompasses the broader institutional and policy environments shaping these interactions. The study suggests that care fragmentation is not just a result of individual behaviors but is rooted in systemic issues such as lack of integrated care pathways, inconsistent communication, and differing institutional priorities. These systemic factors are part of the social practice that professionals must navigate, significantly influencing collaboration effectiveness. Fairclough's framework shows how these systemic issues are reproduced and maintained through discourse, contributing to challenges in achieving cohesive care.

Both Fairclough's and Bourdieu's frameworks illustrate how the challenges of cross‐sectoral collaboration are deeply rooted in social practices and power dynamics within healthcare. Addressing these challenges involves examining behaviors, policies, and the underlying social structures that influence professional thinking, communication, and actions. Fairclough's discourse theory highlights the importance of analyzing and reshaping discourse to transform social practices, suggesting that adopting more inclusive, patient‐centered language may help reduce the divide between hospital and community care.

## 5 Discussion

This research explores the ways in which professionals use language to navigate cross‐sectoral collaboration between mental health hospitals and municipalities, employing a critical discourse analysis grounded in Fairclough's framework (Fairclough 1989). The study offers significant insights into how discursive practices both shape and are shaped by the structural and cultural complexities of healthcare systems. These findings contribute to a deeper understanding of the persistent challenges in achieving integrated, patient‐centered care, particularly in contexts where sectoral boundaries create organizational and professional divides. The findings corroborate existing literature, which highlights the enduring difficulties in achieving seamless integration between hospital‐based and community‐based services. Such challenges are frequently attributed to structural barriers, divergent organizational cultures, and the inherent power imbalances between different sectors (Hersted, Ness, and Frimann 2020; Jørgensen et al. 2023; Jørgensen, Rasmussen, et al. 2022). This study adds to this body of work by demonstrating how language not only reflects these divides but also serves to perpetuate them. Fairclough's discourse analysis framework (Fairclough 1992) illustrates that professionals' communication practices are not merely a reflection of these structural challenges; they actively reinforce or challenge the status quo within cross‐sectoral collaborations.

A key contribution of this research is its focus on the language used by professionals when discussing cross‐sectoral collaboration. Recurring themes such as “treatment plans,” “network meetings,” and “medication management” point to a discourse centered on clinical outcomes and structured processes. This language reflects a medicalized approach to care, deeply ingrained in hospital‐based services (Davidson 2019; Macpherson et al. 2016; Pelletier et al. 2020; Waters et al. 2015; Whitwell et al. 2017). This contrasts with the more holistic, patient‐centered discourse typical of community‐based services, which emphasizes long‐term support and individualized care (Mead and Bower 2000). This dichotomy in discursive practices underscores a broader cultural divide between hospital‐based and community‐based professionals, where the former operates within a framework that prioritizes efficiency, standardization, and protocol‐driven care remove Conversely, community‐based professionals adopt a more flexible approach, focusing on the social determinants of health and long‐term recovery goals (van den Brink et al. 2018). These contrasting discourses create a fundamental barrier to effective collaboration, especially when not properly aligned (Braithwaite et al. 2015).

Fairclough, (1989) concept of discourse power is particularly pertinent here, as it illuminates the mechanisms through which certain professional voices dominate conversations about patient care. Hospital‐based professionals, by virtue of their institutional authority, often exert greater influence over care decisions, overshadowing the contributions of community‐based workers (Foucault 1982). This imbalance is clearly reflected in the development and implementation of care plans, where hospital‐based professionals frequently dictate the terms of care, while community‐based professionals and even patients assume a more reactive role. The study's analysis of transitivity further reveals how these power dynamics are sustained through language. Hospital professionals often adopt an active linguistic position, emphasizing their control over the care process. In contrast, community‐based professionals and patients are frequently positioned in a passive role, portrayed as recipients of decisions made by others. This linguistic construction serves to reinforce the hierarchical nature of healthcare interactions, where decision‐making power is concentrated among certain professional groups, potentially marginalizing the contributions of others. The contribution of this research is its emphasis on patient‐centered care. While there is a rhetorical commitment to involving patients in their own care, the actual language used by professionals often reflects a more controlled, directive approach (Jørgensen, Dahl, and Frederiksen 2020; Jørgensen et al. 2024). This discrepancy suggests a gap between the ideal of patient‐centered care and its practical implementation. Bridging this gap requires that healthcare professionals not only listen to patients' perspectives but also use empowering language that facilitates patients' active involvement in decision‐making. Training in communication techniques such as motivational interviewing (Miller and Rollnick 2002), which emphasizes open‐ended questions and reflective listening, could be instrumental in achieving this goal. Furthermore, care plans should be developed collaboratively with patients, making their personal goals and preferences a focal point of the care process (Coulter and Collins 2011). This research also sheds light on the systemic barriers to effective cross‐sectoral collaboration. The linguistic fragmentation of care delivery, as revealed through this analysis, reflects broader systemic issues that transcend individual interactions. Addressing these challenges requires not only structural changes, such as policy reforms that incentivize collaboration but also cultural shifts within healthcare organizations. Moving away from a siloed approach to care toward a more integrated model requires the development of shared goals and mutual understanding across sectors. Leadership initiatives that model collaborative behavior and interdisciplinary training programs (Miller and Rollnick 2002) can support this cultural shift, fostering an environment that values the contributions of all sectors involved in patient care.

Drawing on Bourdieu's theory of habitus (Bourdieu 1977; Bourdieu 1990), this study also suggests that efforts to foster cross‐sectoral collaboration must extend beyond linguistic structures to encompass the broader practices and dispositions that professionals bring to their work. Habitus shapes not only the language that professionals use but also the actions they take in practice. Understanding how professionals' habitus influences their behavior in collaborative settings is crucial for designing interventions that promote more integrated care. While this study offers significant insights into the role of language in cross‐sectoral collaboration, further research is needed to explore how these discursive practices play out in different cultural and institutional contexts. Future research could focus on how these dynamics vary across healthcare systems in different countries, potentially revealing insights that can inform global best practices for cross‐sectoral collaboration. Additionally, as healthcare increasingly relies on digital platforms for communication, understanding how these technologies influence language and power dynamics in cross‐sectoral collaboration an important area for further exploration is. Research could examine how the design of these tools either facilitates or hinders effective collaboration and how professionals adapt their communication practices in response to the growing use of digital technologies in healthcare.

## 6 Methodical Considerations

While this study provides valuable insights into the discursive practices shaping cross‐sectoral collaboration in mental healthcare, it has several limitations. First, the study is based on a relatively small and localized sample of professionals from a specific region in Denmark, which may limit the generalizability of the findings to other contexts or healthcare systems. Additionally, the focus on discourse analysis means that the study primarily interprets language and communication patterns without directly measuring the outcomes of these interactions on patient care. This reliance on discourse analysis may also introduce subjective interpretations, as the analysis is influenced by the researchers' perspectives and the specific theoretical framework employed.

Moreover, the study does not capture the perspectives of patients or community‐based workers directly, which could have provided a more comprehensive understanding of the collaborative dynamics. The cross‐sectional nature of the study limits its ability to assess changes in discourse and collaboration practices over time. Finally, while the study highlights power dynamics and communication barriers, it does not provide detailed recommendations for practical interventions, leaving room for further research to explore specific strategies for improving cross‐sectoral collaboration in mental healthcare.

## 7 Clinical Implications

The results of this research have several important implications for the future of cross‐sectoral collaboration in mental healthcare. Firstly, the study underscores the need for healthcare systems to address the entrenched power dynamics that can impede effective collaboration. By recognizing and actively working to balance these dynamics, professionals can foster a more inclusive and patient‐centered approach to care. This involves not only changes in communication practices but also the adoption of policies and training programs that promote shared decision‐making and respect for diverse professional perspectives.

Additionally, the study highlights the importance of creating integrated care pathways that streamline transitions between hospital and community‐based services. These pathways should be supported by clear protocols and regular interdisciplinary meetings to ensure continuity of care and alignment of treatment goals across sectors. The findings also suggest that healthcare systems need to prioritize the development of a shared language and understanding among professionals from different disciplines and sectors, which can help bridge gaps in care and improve patient outcomes. Overall, the research indicates that more systemic changes are required to move toward a truly collaborative and patient‐centered model of mental healthcare, which could lead to better health outcomes and more efficient use of resources.

## 8 Conclusion

This study provides a comprehensive analysis of how language influences cross‐sectoral collaboration between mental health hospitals and municipalities. Through the application of Fairclough's critical discourse analysis framework, the research reveals that discursive practices play a crucial role in maintaining or challenging the structural and cultural divides that complicate effective collaboration. The findings highlight the dominance of a medicalized discourse in hospital settings, which often marginalizes the more holistic, patient‐centered approaches prevalent in community‐based care. This discursive dominance perpetuates power imbalances, with professionals frequently positioned as the primary decision‐makers, while patients and community‐based workers are relegated to more passive roles.

The study also underscores the challenges of implementing patient‐centered care within these collaborative frameworks. Despite rhetorical commitments to involving patients in their care, the language used by professionals often reflects a more controlled and directive approach, limiting genuine patient empowerment. These findings suggest that for true integration and collaboration to occur, there needs to be a conscious effort to develop a shared language that values and incorporates the perspectives of all stakeholders, particularly those of patients. In addition, an awareness of the connection between spoken and written language and action in practice is an important field of attention. Addressing these discursive barriers requires targeted interventions in communication practices, the development of integrated care pathways, and a shift toward a more inclusive, patient‐centered model of care. By fostering a culture of collaboration and mutual respect across sectors, healthcare systems can better meet the needs of patients and improve overall care outcomes. This study contributes valuable insights into advancing these goals, offering a path forward for more effective and equitable cross‐sectoral collaboration in mental healthcare.

## Ethics Statement

The research adhered to ethical standards in scientific inquiry, receiving approval from the Research Ethics Committee (Institutional Review Board) at Roskilde University, Denmark, under reference number KJ‐03.23. The study complied with the principles outlined in the Helsinki Declaration (The Ministry of the Interior and Health 2024; World Medical Association 2013). As the research did not aim to exert physical or psychological influence on participants, formal permission from a biomedical ethics committee was deemed unnecessary. Participants were fully briefed on the project and provided both written and verbal consent to participate. The principal investigator explained the study's objectives and ensured participants understood their legal and ethical rights. Approval to collect empirical data was obtained from a manager at the Regional Zealand mental health organization. All invited participants willingly consented, and there were no withdrawals from the study.

## Conflicts of Interest

The authors declare no conflicts of interest.

## Data Availability

Data supporting this study's findings are available on request from the corresponding author. The data are not publicly available due to privacy or ethical restrictions.
